# CD133 Role in Oral Carcinogenesis

**DOI:** 10.31557/APJCP.2020.21.9.2501

**Published:** 2020-09

**Authors:** Ealber Carvalho Macedo Luna, Thâmara Manoela Marinho Bezerra, Paulo Goberlânio de Barros Silva, Roberta Barroso Cavalcante, Fábio Wildson Gurgel Costa, Ana Paula Negreiros Nunes Alves, Filipe Nobre Chaves, Karuza Maria Alves Pereira

**Affiliations:** 1 *Department of Dental Clinic, Division of Oral Pathology, Faculty of Pharmacy, Dentistry and Nursing, Federal University of Ceará, Fortaleza, Brazil. *; 2 *Department of Dental Clinic, Faculty of Pharmacy, Dentistry and Nursing, Federal University of Ceará, Fortaleza, Brazil. *; 3 *Department of Oral Pathology, Faculty of Dentistry, Universidade de Fortaleza, Fortaleza, Ceara, Brazil. *; 4 *School of Dentistry, Federal University of Ceará Campus Sobral, Sobral, Brazil. *; 5 *Departament of Morphology, School of Medicine, Federal University of Ceará, Fortaleza, Brazil. *

**Keywords:** premalignant lesions, oral squamous cells carcinoma, oral carcinogenesis, CD133, stem cells

## Abstract

**Objective:**

to investigate CD133 immunoexpression, cancer stem cells marker, in oral epithelial dysplasias (OEDs) and oral squamous cells carcinomas (OSCCs) and understandits possible involvement in the malignant transformation process of these lesions and to better elucidate their biological behavior.

**Material and methods:**

Tissue samples of 15 cases of OSCCs and 15 OEDs were subjected to CD133 antibody immunohistochemistry reactions. The analysis used quantitative parameters (number of immunostained cells regardless of immunostaining sublocations).

**Results:**

All samples of OSCCs and OEDs showed positive immunostaining, with no significant difference between these groups (p = 0.283). We did not observe statistical difference between the degree of dysplasia and the amount of CD133+ cells (p = 0.899). CD133 immunoexpression showed no association with the OEDs and OSCCs sites. It was observed that nuclear and cytoplasmic immunostaining was more evident with the progression of the malignant process.

**Conclusion:**

It is suggested that the CD133 cellular localization together with the histopathological criteria of OEDs classification can contribute to provide more concrete indications about the oral carcinogenesis process.

## Introduction

Oral cancer has been gaining worldwide attention for being the 11th most common carcinoma around the globe (D’Souza and Addepalli, 2018). In Brazil, more than 14 thousand new cases of this disease are estimated for the 2018-2019 biennium, being the 5th most common cancer in me n (INCA,2018). This incidence represents 2.6% of all registered cancers in Brazil, one of the highest in the world and of significant expressiveness in Latin America (INCA, 2018). Approximately 90% of oral cancers are Oral Squamous Cells Carcinomas (OSCCs) (Tandon et al., 2017; D’Souza and Addepalli, 2018). The etiological base of oral cancer is tobacco intake, smoking, smokeless tobacco (snuff or chewing tobacco), alcohol and areca nut intake, excessive sunlight exposure, reverse end smoking and Human Papilloma Virus (HPV) (D’Souza and Addepalli, 2018).

Cancer in the oral cavity is often preceded by a precursor lesion (Ganesh et al., 2018) termed Potentially Malignant Disorders (PMDs). In general, the causes or risk factors for PMDs are the same for OSCCs (Porter et al., 2018). However, not all PMDs will undergo malignant transformation, requiring histopathological findings of Oral Epithelial Dysplasia (OED) in histopathological examination. OEDs have been categorized into three major sections: minimum, moderate and critical (D’Souza and Addepalli, 2018), with higher chances of malignant transformation in the last two of them. However, there is no molecular or even histopathological pathognomonic hallmark that can predict malignant transformation of PMDs (Ganesh et al., 2018). 

Recent research suggests that Cancer Stem Cells (CSC) hold the key to unlocking effective strategies to curb initiation and growth of several malignant neoplasms (de Moraes et al., 2017), including OSCCs (Saluja et al., 2019). The CSCs are identified by some surface markers, among them CD133, also called Prominin-1. CD133 consists of an N-terminal extracellular domain, five transmembrane domains, and an intracellular cytoplasmic tail with functional tyrosine kinase sites (Udeabor et al., 2012), which will interact with distinct cytoplasmic partners, regulating signal molecules and changing the cancer metabolism, thus promoting the CSC properties (Jang et al., 2017).

The identification of CSCs in OEDs and OSCCs may help in understanding the role of CSCs in the oral carcinogenesis process. It is expected that CSC biomarkers can be used together with histopathological parameters indicative of malignancy in order to contribute to a more accurate diagnosis of the risk of malignant transformation, since the association between the degree of oral dysplasia and malignant transformation remains debatable (Speight et al, 2018). Thus, this research aimed to evaluate CSCs participation, through CD133 immunoexpression, in process of oral carcinogenesis through OEDs of different degrees and OSCCs. 

## Materials and Methods

This study consisted of an observational, analytical, and cross-sectional study, using the diagnosis and immunomolecular analysis of malignant and premalignant lesions. We analyzed 15 cases of OEDs and 15 of OSCCs (size of the sample compatible with the annual demand of patients in the service). All samples were embedded in paraffin and obtained from incisional biopsies from patients of the Stomatology Clinic of the Federal University of Ceará, Sobral Campus. Samples were collected from October 2013 to October 2014. 

*Histomorphometric analysis*

Specimens were fixed in 10% formalin, embedded in paraffin, sectioned at 5µm, stained with hematoxylin–eosin and mounted on glass slides for histopathological analysis. OEDs specimens were classified according to WHO classification (Barnes et al, 2005). The results of this classification were as follows: 11 were mild dysplasia, 02 were moderate, and 02 were severe.

*Immunohistochemical reaction*

For immunohistochemistry, 3µm sections were cut from paraffin embedded material. All tissue samples were processed using standard methods and serial sections were used for immunohistochemical reaction (IHC). After deparaffinization and rehydration, slides were subjected to heat-induced epitope retrieval (citrate in pH 6,0, for 30 minutes at 99ºC) in a Pascal water bath (DakoCytomation). Endogenous peroxidase activity was blocked for 30 min with 0.3% hydrogen peroxide followed by 1% protein blocking for 10 min. The sections were incubated with primary antibodies anti-CD133 (GTX60471, GeneTex^®^, San Antonio, TX, USA) for 90 minutes, at room temperature, in the dilution of 1:650. The samples were then incubated with the secondary antibody LSAB Kit (DAKO^®^, Carpentaria, CA, USA) for 10 min at room temperature. Next, development was performed using a chromogen solution prepared with DAB (3-30-diaminobenzidine) for 5 min in a dark chamber (DAKO^®^, Carpentaria, CA, USA) and Harris hematoxylin was used for counter staining. Finally, coverslips were placed on the samples on glass slides, which were examined under a Leica DM 2000 optical microscope. A positive control (breast carcinoma) was included in each reaction along with the samples. A negative control lacking primary antibody was performed in parallel with incubation of the experimental samples.

*Quantitative analysis*

Quantitative analysis of CD133 glycoprotein expression was performed by percentage of stained cells in 5 random areas as examined in X400 magnification using the software Image J (Image and Processing Analysis in Java – Rasband, W.S., ImageJ, National Institutes of Health, Bethesda, Maryland, USA, http://rsb.info.nih.gov/ij/, 1997-2004) (Adapted from Ravindran and Dervarage, 2012). In case of disagreement, the slides were re-evaluated by the 2 observers until a consensus was reached. 

*Statistical analysis*

The analysis of CD133 positive cells was submitted to the Kolmogorov-Smirnov normality test, expressed as mean ± standard error of the mean (parametric data) and compared between groups by Student’s t test and ANOVA followed by the Bonferroni post-test. Significance index p <0.05 was adopted for all evaluations performed in GraphPad Prism version 5.0 software for Windows®.

## Results

The sample consisted of 30 cases, 15 OED and 15 OSCC samples. The clinical characterization (clinical profile) of the studied sample was performed. Of the 15 cases of OSCC, it was evidenced that males were the most affected, with 60% of the cases. Age ranged from 33 to 83 years, with a mean age of 50.8 years, and more prevalent in the fourth and fifth decades of life (66.6% of cases). The tongue region was the most prevalent anatomical site, affecting approximately 40% of the cases. The analysis performed in the 15 cases of OED showed that the female sex was the most prevalent (73.3%). Age ranged between 17 and 85 years, with most cases in the seventh decade of life (33.3%). The region of jugal mucosa was the most frequent site of involvement, corresponding to 33.3% of the OED cases analyzed.

**Figure 1:**
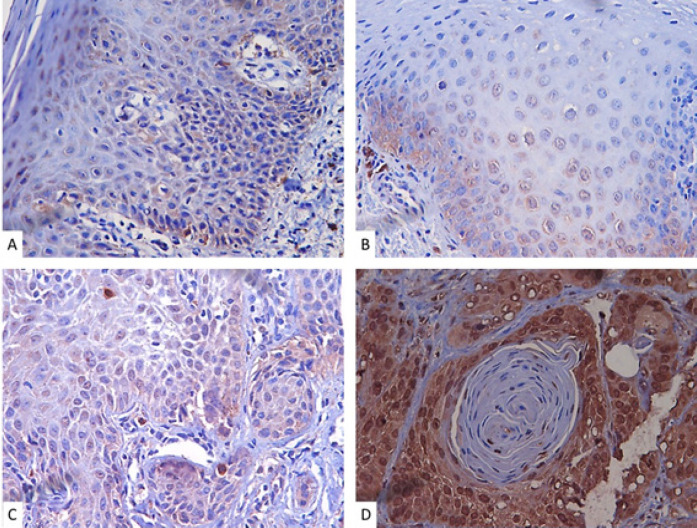
Immunostaining for CD133 in the OED and OSCC Samples Analyzed. A, B and C, respectively, show cases of mild, moderate and severe dysplasia. A: Mild dysplasia showing CD133 positive cells in the cytoplasm with rare nuclear immunostaining (Magnification 400x). B: Moderate dysplasia showing both types of immunostaining (cytoplasmic and nuclear) (Magnification 400x). C: Severe dyspasia showing greater number and intensity of cellular immunostaining for CD133 in relation to the other degrees of dysplasia (both cytoplasmic and nuclear immunostaining are observed) (Magnification 400x). D: Example of immunostaining in OSCC. Note that the immunostaining intensity and number of CD133+ cells is greater than in cases of dysplasia. There is a predominance of nuclear immunostaining when compared to cases of dysplasia (Magnification 400x)

Immunohistochemical analysis of CD133 revealed nuclear and cytoplasmic immunostaining in OEDs and OSCCs of all evaluated specimens. Interestingly, nuclear immunostaining as well as intensity increased according to the progression of the malignancy process (Figure 1 A, B, C and D).

Regarding the immunostaining profile, CD133 positive expression was observed in all samples. The CD133 immunoexpression was 82.6 ± 7.2 and 77.6 ± 16.0 in patients with OSCCs and OEDs, respectively. However, there was no statistically significant difference between the groups studied (p = 0.283) (Graphic).

Regarding the degree of dysplasia, it was observed that 78.0 ± 18.4 of epithelial cells showed positive immunostaining in cases of mild dysplasia, while 72.7 ± 11.4 and 80.1 ± 1.8 showed positive marking in cases of moderate and severe dysplasia, respectively, with no difference statistically significant among the three groups (p = 0.899) (Graphic 2).

Regarding anatomical location, the percentage of immunostaining for CD133 showed no association with the different sites: tongue (OED: 69.6 ± 23.2 and OSCC: 83.5 ± 9.3) (p = 0.217), palate (OED: 74.5 ± 6.7 and OSCC: 86.8 ± 10.3) (p = 0.193), buccal mucosa (OED: 84.8 ± 14.7 and OSCC: 79.0 ± 5.7) (p = 0.618) and mouth floor (OSCC: 82.3 ± 4.4) (p = 0.193) (Graphic 3). There were no cases of OED on the floor of the mouth.

The percentage of immunostaining for CD133 in relation to sex did not show any statistically significant results: male sex (OED 76.4 ± 10.9 and OSCC 82.9 ± 6.3) (p = 0.526) and female sex (OED: 78.0 ± 17.9 and OSCC 82.1 ± 8.9) (p = 0.588) (Graphic 4).

**Graphic 1:**
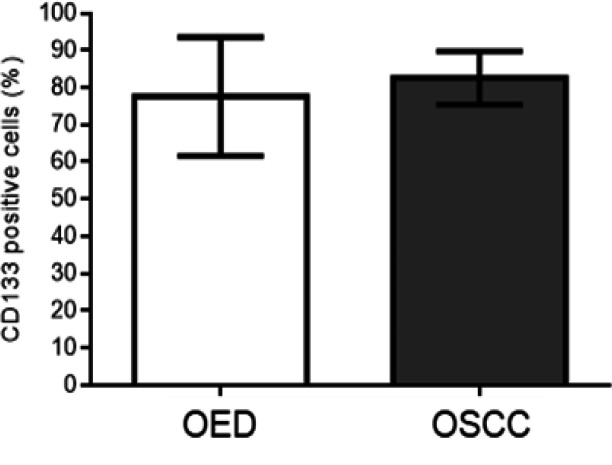
Immunostaining Profile of OED and OSCC Cases. There was no statistically significant difference between the groups (T-test unpaired. p = 0.283)

**Graphic 2:**
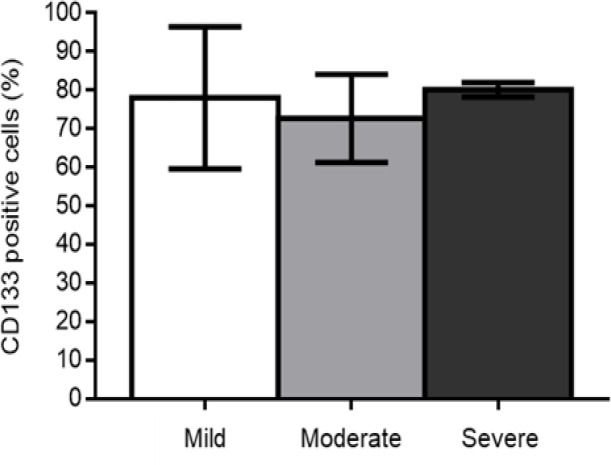
Immunostaining Profile of Dysplasia Degree. There was no statistically significant difference between the groups (One-way ANOVA test. p = 0.899)

**Graphic 3:**
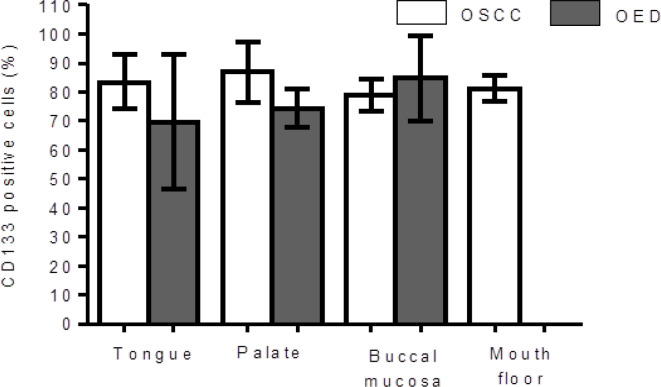
CD133 Positive Cells Showed No Association with the Different OED and OSCC Sites. (One-Way ANOVA Test)

**Graphic 4:**
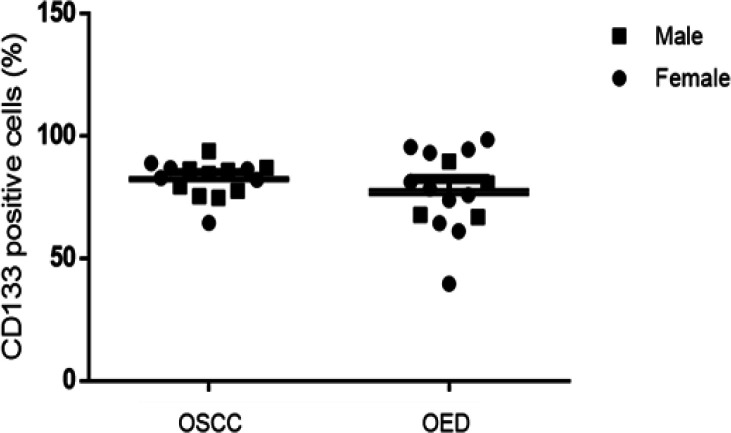
There Was No Statistical Difference Regarding CD133 Immunostaining in OEDs and OSCCs Cases when Comparing the Patients' Dgenders. (Two-way ANOVA test)

## Discussion

This research found a higher OSCC prevalence among males (60%). The highest incidence of oral cancer in men is demonstrated in several studies (Oliveira et al., 2015, Tandon et al., 2017). This may be because of the greater exposure of men to the risk factors. In addition, gender differences in oral cancer may reflect different cultural behavior and lifestyle factors. The occurrence of oral cancer increases with age in all parts of the world. We detected that the 4^th^ and 5^th ^decades of life were the age groups most affected by OSCCs. However, an alarming increase in the incidence of oral cancers among the younger adults has been reported. This happens due to an increase in the usage of tobacco (smoked or chewed) in young adults in comparison to older individuals (Abdulla et al., 2018). Other risk factors that lead to young patients OSCC development include influence of environmental carcinogens, stress and viral infections. The oral tongue was the region most affected by OSCC in this study similarly to other studies (Rivera, 2015). The predilection for this region may be associated with the pooling of carcinogens in saliva, creating risk zones (Brandizzi et al., 2008). However, geographic differences may be related to the intraoral distribution of OSCC. This happens, for example, in India, where most cases of OSCCs affect the buccal mucosa (Tandon et al., 2017) due to tobacco use, particularly chewing.

Among the cases of OED analyzed, most of them affected the female sex (73.3%). PMDs are less common in females, however, when present, they have a higher risk for malignant transformation. It is still unclear why women are more predisposed to malignant transformation compared with men (Speight et al., 2018). Most of the OED patients were in the 7^th^ decade of life in the present study. In a large Swedish study, the highest malignant transformation rate was found in those with age of 70-89 years (Napier et al., 2003). Therefore, special attention should be given to patients with OEDs in this age group. The region of jugal mucosa was the most frequent site of involvement in this study. The oral tongue and the floor of the mouth are the sites of major involvement of the OEDs (Gandara-Vila et al., 2018; Speight et al., 2018), and are also considered anatomical sites of high risk for malignant transformation, this location is almost always related to etiologic factors and therefore may vary by geo Figure location and local habits (Speight et al., 2018).

Recent experimental evidence shows that oral cancer is initiated through CSCs, which play a crucial role in cancer malignant progression, therapeutic resistance and recurrence (Baillie et al., 2017). Oral CSCs have been isolated in various forms, among them using specific biomarkers. CD133 is a marker that has been gaining popularity for OSCC identification and it was first described as a hematopoietic stem cell marker (Ravindran and Devaraj, 2012), but currently it can be considered a CSC marker of several solid tumors, such as breast, gastric, pulmonary, hepatic, prostate, pancreas and thyroid (Okamoto et al., 2013; Gao et al., 2014; Yu et al., 2015;).

However, a single biomarker is not able to unambiguously identify CSCs, since it is likely that there is an overlapping hierarchy of subsets of CSC populations (Baillie et al., 2017). Consequently, most of the studies that investigate and characterize CSCs use a combination of biomarkers of this cellular type (Baillie et al., 2017). The markers already described for CSCs of OSCCs are (OCT4, NANOG, SOX2, STAT3, CD44, CD24, Musashi-1, ALDH, components of the renin–angiotensin system, CD29) (Liu et al., 2013; Baillie et al., 2017; de Moraes et al., 2017). We chose CD133 to identify oral CSCs because it is more highly expressed in the CSC population compared to the parental normal population (Pozzi et al., 2015). In addition, although CD133+ CSCs are present in OSCCs, these cells are preferentially expressed in colon, brain, and lung cancer (de Moraes et al., 2017), with little information on the role of CD133+ CSCs in oral carcinogenesis.

We found that all cases of OSCCs and OEDs showed CD133 positive marking, with a large percentage of cells immunopositive for both lesions (Figure 1). However, although these cells are the foundation of tumorigenesis, they represent only a small portion of tumor cells (Wang et al., 2016). The frequency of CSCs CD133+ appears to be variable among OSCCs and Head and Neck Squamous Cell Carcinoma (HNSCCs) with cases of low (Wang et al., 2016; de Moraes et al., 2017) and high cell CD133+ expression (Ravindran and Devaraj, 2012; Liu et al., 2013; Manelli et al., 2015). We believe that these conflicting results occur because the tumor samples have different degrees of malignancy and clinical outcome, since the presence of CSCs have been related to tumors with worse prognosis, recurrent disease, treatment failure and metastasis (Satpute et al., 2013; Jang et al., 2017). Research with OSCCs shows that stage III and IV tumors have higher amounts of CD133+ CSCs (Singh et al., 2018). In addition, the localization of overexpressed CD133 at nucleus and cytoplasm is related to poor prognosis (Huang et al., 2015). We detected high cytoplasmic and nuclear expression in the OSCC samples (Figure 1), which may also explain why our results are diverging from some studies.

We did not detect statistically significant differences in CD133 immunoexpression between OSCCs and OEDs (Graphic 1) nor between different degrees of OEDs (Graphic 2). However, it is quite evident that the marking pattern changes between the different lesions, with a higher nuclear and cytoplasmic marking in the more advanced cases of carcinogenesis (Figure 1A, 1B, 1C and 1D). The release of CD133 from the plasma membrane into the cytoplasm is related to the uptake of glucose under conditions of deprivation (Jang et al., 2017). Therefore, CD133 signaling in the cytoplasm is likely to potentiate the survival of tumor cells under conditions of nutrient restriction or stress (Jang et al., 2017), conditions known to be linked to the oral carcinogenesis process. Research shows that CD133+ CSCs are related to OSCCs of worse staging point to the involvement of CD133+ CSCs in the process of transformation of premalignant oral lesions (Ravindran and Devaraj, 2012; Liu et al., 2013). In addition, CSC CD133+ are present in most OEDs that have undergone malignant transformation for OSCCs (Liu et al., 2013). Thus, CD133 serves as a predictor to identify oral premalignant lesions with a high risk of oral cancer development. 

We found no difference in expression between CD133 and the localization sites of OSCCs and OEDs (Graphic 3). Similar results were also not observed in other studies (Ravindran and Devaraj, 2012; Liu et al., 2103), suggesting that the expression of this protein is independent of the location of the lesion in the oral cavity. This is probably because the anatomical locations covered in this study (tongue, palate, buccal mucosa and buccal floor) are susceptible to the same etiologic factors of carcinogenesis (tobacco smoke and alcohol consumption). 

This study has limitations like any other purely immunohistochemistry research, which limited the authors to realize greater conjectures. In addition, despite the size of the sample match the sample calculation, we believe that more and more homogeneous sample could modify the results. 

In conclusion, the presence of multiple CSC subtypes within OSCCs making investigation of these cellular types reliant on the use of multiple markers. This study has limitations because it uses only CD133 as a biomarker to identify oral CSCs, but provides important evidence on the cellular location of CD133 to be linked to the oral carcinogenesis process. Thus, despite the histologic grading of OED currently being the most important indicator for determining the risk of malignant transformation, its histologic classification may involve subjectivity. Thus, the cellular localization of CD133 may provide evidence of development of CSCs. This tool, along with the histopathological findings, may better identify premalignant oral lesions with increased chances of malignancy.
